# Determination of pesticide residues in rooibos (*Aspalathus linearis*) teas in South Africa

**DOI:** 10.1016/j.toxrep.2022.04.001

**Published:** 2022-04-15

**Authors:** O.M. Areo, J.O. Olowoyo, L.S. Sethoga, O.A. Adebo, P.B Njobeh

**Affiliations:** a Department of Biotechnology and Food Technology, Faculty of Science, University of Johannesburg, P.O. Box 17011, Doornfontein Campus, 2028 Gauteng, South Africa; b Department of Biology and Environmental Sciences, Sefako Makgatho Health Sciences University, School of Science and Technology, P.O. Box 139, Pretoria 0204, South Africa; c Department of Chemistry, Sefako Makgatho Health Sciences University, School of Science and Technology, P.O. Box 139, Pretoria 0204, South Africa

**Keywords:** Pesticide residues, Quick easy cheap effective rugged and safe, Limit of quantification, Maximum residue limits, South Africa

## Abstract

An efficient gas chromatography–mass spectrometry approach was used in this study to quantify 13 pesticide residues in rooibos teas purchased from registered retail outlets in South Africa between November 2019 and April 2020. A QuEChERS (Quick, easy, cheap, effective, rugged, and safe) procedure was used to extract pesticides using 7.5 mg of graphitized carbon black (GCB), 50 mg of primary secondary amine (PSA), and 150 mg of anhydrous MgSO4. In order to compensate for the matrix effect, matrix matched calibration curves ranging from 10 µg/kg–500 µg/kg were applied for accurate quantification. For validation purposes, accuracy tests were conducted using a blank tea sample spiked with pesticide standards at two different concentrations (10 and 100 μg/kg). Most of the analytes were recovered within acceptable recovery ranges (72–106%), with a relative standard deviation of less than 20%. The limits of quantification were low, all falling below 10 μg/kg which meets the maximum residue limits (MRLs). The validated method was used to analyze 100 tea samples, and among the pesticides analyzed, deltamethrin and lambda-cyhalothrin were detected in only one samples at a concentration (92.11 and 66.41 μg/kg, respectively) below the MRLs stipulated by the European Union. The level of pesticides that are commonly used in tea should be checked often.

## Highlights

• Rooibos teas, a popular beverage in South Africa.
• QuEChERS methods reduced matrix effect in multi-pesticide analysis.
• 13 pesticide were determined simultaneously by GC-MS-SIM.
• GC-MS-SIM demonstrated high sensitivity and confirmatory capacity needed for determining pesticide residue at low levels.
• The validated method was applied successfully to rooibos tea samples from South Africa.

## Graphical Abstract

**Figure:**
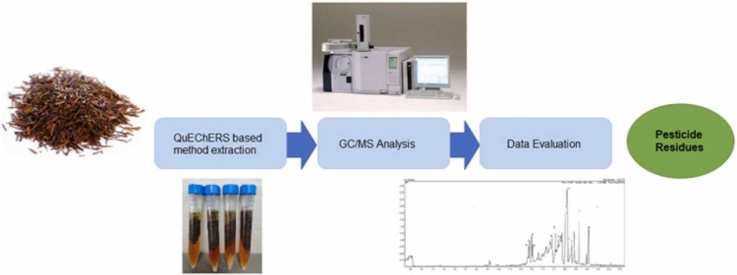

## 1 Introduction

Tea is among the most widely consumed beverages worldwide [10], [9], [11]. In South Africa, rooibos is one of the most common drinks, with its medicinal properties having been discovered [24], [47]). It is a caffeine-free tea made from the leaves of a South African plant (*Aspalathus linearis*), popularly grown in the Western Cape [24]. Rooibos tea was recently approved for registration as an African food under the status of international protection by the European Union [30]. This addition will help to maintain the long-standing link between rooibos and South Africa. According to the South African Rooibos Council [38], about 31 million South African tea drinkers believe rooibos is the most popular tea. Numerous studies have reported tea to have significant anticarcinogenic, antioxidant, thermogenic, antimicrobial, anti-inflammatory, and probiotic properties [6], [24], [31].

Tea is invaded by a variety of pests and diseases, and it is important that plant protection agents be used to mitigate the degree of the attack. Insect pests play a major role in lowering the quality and quantity of tea production. As a result, tea producers use a variety of pesticides to tackle these issues in order to maximize output and economic benefits [33]. A large-spectrum synthetic chemical pesticides such as carbamates, organophosphates, neonicotinoids, synthetic pyrethroids and zimidazoles, are used widely for tea pest control [13], [33]. Various investigations have been conducted on pesticide residues in various teas and their leaching behavior during brewing [8], [21], [5], [29]. As found, it has been concluded that any residues that remain on dried tea leaves may diffuse into tea infusions, potentially exposing consumers to dangerous chemicals [36], [1]. Pesticides accounts for a number of human health issues, ranging from acute symptoms like nausea and headaches to long-term effects like tumors, infertility, endocrine disruption, and birth defects [7], [2]. International agencies such as the European Union (EU), The Food and Agriculture Organization (FAO), and the United States Environmental Protection Agency (EPA) have developed and proposed maximum residual limits (MRLs) for various pesticides in tea. The European Union (EU) Pesticides database [16] presently provides 486 pesticide residues in the tea MRL list, based on Regulation (EC) No 396/2005 [14] (Table 1).

**Table 1:** Physiochemical properties of the selected pesticides and their maximum residue limit [16].

Compound	Action mode	Molecular Formula	Molecular weight	MRL (mg/kg)
Pirimiphos-methyl	Insecticides	C_11_H_20_N_3_O_3_PS	305.34	0.05
Fenitrothion	Insecticides	C_10_H_15_0_3_PS_2_	278.3	0.05
Malathion	Insecticides	C_10_H_19_0_6_PS_2_	330.4	0.5
Chlorpyrifos	Insecticides	C_9_H_11_C_l3_NO_3_PS	350.6	2
Endosulfan	Insecticides	C_9_H_6_Cl_6_O_3_S	406.92	30
Ethion	Insecticides	C_9_H_22_O_4_P_2_S_4_	384.5	3
4,4, DDE	Insecticides	C_14_H_8_Cl_4_	318.02	0.2
Deldrin	Insecticides	C_12_H_8_C_l6_O	380.9	0.02
Triazophos	Insecticides	C_12_H_16_N_3_O_3_PS	313.31	0.02
4,4, DDT	Insecticides	C_14_H_9_Cl_5_	354.5	0.2
Deltamethrin	Insecticides	C_22_H_19_Br_2_NO_3_	505.2	5
Bifenthrin	Insecticides	C_22_H_22_CIF_3_O_2_	422.9	30
Lambda-Cyhlaothrin	Insecticides	C_23_H_19_CIF_3_NO_3_	448.8	1

The MRL for various pesticides in tea, on the other hand, varies depending on national laws in tea-exporting countries. To put it another way, MRLs of pesticide residues in different countries where tea is grown are not harmonized [17]. As a result, it is vital to develop effective analytical methods for monitoring pesticide levels in tea. Due to the low levels of pesticide residues in tea samples and a high number of co-extracted components that limit the efficacy of analytical methods, it is quite challenging to investigate pesticide residues in complex samples like teas [29]. The presence of high amounts of caffeine, pigments, and polyphenols is the main reason for the tea matrix's complexity [18].

Pesticide residues have nonetheless been determined using a variety of developed and optimized methods, including solid-phase extraction, quick solvent extraction, and solid-phase micro-extraction [46]. Furthermore, most of these approaches are time-consuming and necessitate huge amounts of solvents [20]. The matrix effect is not mitigated by solvent extraction or other traditional analytical methods [22]. Sample preparation is one of the most critical components of the analytical process, which must be fast, dependable, succinct, and environmentally sustainable. The QuEChERS (Quick, easy, cheap, effective, rugged, and safe) method for determining pesticide residues in food samples has already been shown to be effective Kolberg et al., 2011; Park et al., 2011; Lozano et al., 2012; Jeong et al., 2012; Amaraweera and Wickramasinghe , Pitoi et al., 2019; Ly et al., 2020; Tan et al., 2021; Lin et al., 2022[25], [34], [28], [23], [3], [35], [29], [42], [27]. In this study, a modified and validated QuEChERS multi-analyte method using dispersive solid-phase extraction (d-SPE) was adopted and used in conjunction with gas chromatography–tandem mass spectrometry to determine 13 pesticides in 100 different teas.

## 2 Materials and methods

### 2.1 Materials, chemicals, and reagents

**Fig. 1:**
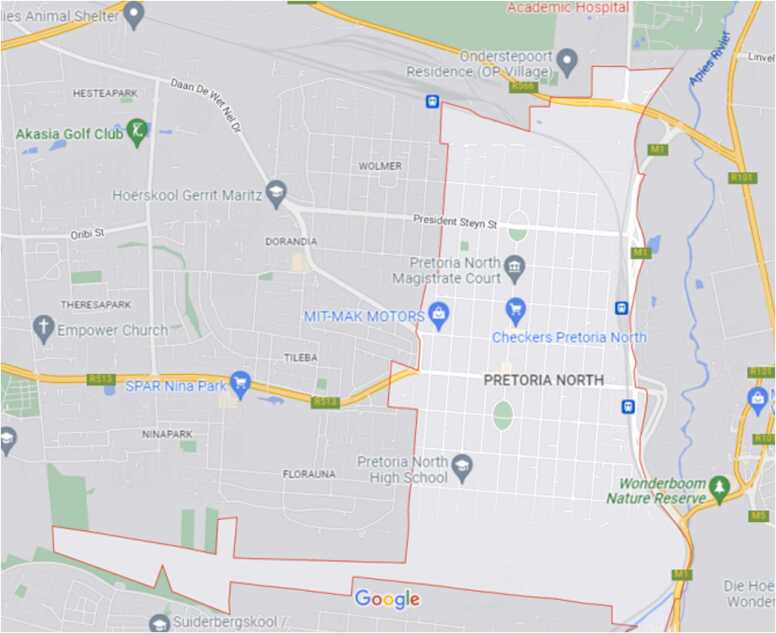
Pretoria North in Gauteng Province, South Africa with the GPS coordinates being 25°40′23″S 28°10′24″E.

A random sampling was performed at Pretoria North (Fig. 1) in Gauteng Province, South Africa with the GPS coordinates being 25°40′23″S 28°10′24″E. A total of 100 samples of rooibos tea were purchased, comprised of natural rooibos teas (n = 12), flavored rooibos teas (n = 36), herbal rooibos teas (n = 32), and ordinary teas (n = 20) during November 2019 and April 2020. After purchase, each sample of tea was coded to obscure its identity and origin. The tea bags were all stored at room temperature in dry, air-tight ziplock bags, until analysis. All the pesticide standards used in this study were bought from Sigma-Aldrich in South Africa. The standard pesticides had purities ranging from 95% to 99.9%. All standard solutions were stored in accordance with the manufacturer's recommendations. Acetonitrile (Chromatographic grade) was purchased from Sigma Aldrich, South Africa, and deionized water (18 MΩ cm) was filtered using the Milli-Q water purification system (Millipore, Bedford, MA). A QuEChERS buffer-salt mixture was purchased from Restek, South Africa, and each portion consisted of 0.5 g of disodium hydrogen citrate sesquihydrate (DHS), 1 g sodium chloride (NaCl), 1 g trisodium citrate dehydrate (TSCD), and 4 g of anhydrous magnesium sulfate (MgSO_4)_. A dispersive solid-phase extraction (dSPE) salt mixture was obtained from the same manufacturer and consisted of 150 mg of anhydrous magnesium sulfate (MgSO_4_), 50 mg of primary secondary amine (PSA), and 7.5 mg of graphitized carbon black (GCB). In acetonitrile, individual stock solutions of 500 μg/kg were prepared for each pesticide standard. Thirteen working standards were prepared using the serial dilution process for the above-mentioned reference pesticide standard at different concentrations (10, 25, 50, 100, 250, and 500 μg/kg).

### 2.2 Sample extraction procedure

Pesticides in tea samples were extracted using the standardized QuEChERS method (EN 15662). In a 50 mL centrifuge tube, 2 g of sample was measured, 10 mL of water was added to hydrate the tea powder, and the tube was left for 30 min before being filled with 10 mL of acetonitrile containing 1% acetic acid. Salting-out was done by adding 1 g TSCD, 0.5 g DHS, and 4 g MgSO_4_, as well as 1 g NaCl were added, and the mixture was vortexed for 1 min before being centrifuged in a TDL-5-A low-speed centrifuge (Anke, Shanghai, China) for 5 min at 4500 rpm. An aliquot of the organic phase (2 mL) from each sample was transferred into a 2 mL centrifuge tube containing a dSPE salt mixture (7.5 mg GCB, 50 mg PSA, and 150 mg anhydrous MgSO_4_). The tube was vortexed for 1 min before being centrifuged for 5 min at 4500 RPM. Then, the clean extract was filtered through a 0.22 µm syringe filter, transferred to a glass vial, and finally subjected to GC-MS analysis.

### 2.3 Instrumentation

The study was conducted using a Shimadzu GC-MS-QP2010Plus gas chromatograph with an OCI/PTV-2010 (on column/Programmable Temperature Vaporization Injector), SPL-2010Plus (Split/splitless injector), and Optic-4 (Multi mode Injector). For data collection and processing, as well as statistics, the GC was connected to an AOC-20i+ s auto injector and auto sampler, as well as a PC running GC-MS solution software and Insight software (Shimadzu Corporation, Kyoto, Japan). The Rxi-5Sil MS capillary column (30 m x 0.25 mm i.d., 0.25 m film thickness) was used as the analytical column. The mass spectrometer was operated in the selective ion monitoring (SIM) mode. The GCMS-QP2010Plus column was set at a linear velocity of 53.9 cm/sec using helium as the carrier gas. The column temperature was programmed as follows: 40 ◦C held for 1 min, at 30 ◦C min^−1^ to 150 ◦C, at 6 ◦C min^−1^ to 200 ◦C, at 16 ◦C min^−1^ to 280 ◦C held for 4.07 min; carrier gas was helium; purity ≥ 99.999%; flow rate 1.0 min^−1^ injection port temperature 250 ◦C; injection volume 1 µL; The total run time was 21.40 min. The temperature of the GC–MS interface was set to 280 °C and the injection volume was 0.5 µL in a splitless mode. The following mass spectrometric settings were used: electron impact ionization mode with a 70-eV ionizing energy, 200 °C ion source temperature, 230 °C interface temperature, and 250 °C injector temperature. To get the fragmentation spectra of the analytes, full-scan data in the *m/z* 50–700 range was obtained, and each compound's selected ion monitor mode was set to one target ion and two qualifying ions (Table 2). Peaks were detected using their retention times and mass spectra after scanning the total ion chromatogram for mixed stock standard solutions. For quantification, the most abundant ion was chosen since it had the greatest signal-to-noise ratio and no chromatographic interference.

**Table 2:** The retention time, monitored ion and selected confirmation ion for the target pesticides.

No	Compound	Ret. Time	Monitored ion	Confirmation Ion
1	Pirimiphos- methyl	12.70	290, 275, 305	290.05
2	Fenitrothion	12.76	277, 125, 109	125.00
3	Malathion	13.00	173, 158, 125	125.00
4	Chloropyrifos	13.14	314, 197, 125	197.00
5	Endosulfan	14.83	125, 167, 225	125.05
6	Ethion	16.05	231, 239, 384	231.90
7	4,4, DDE	15.28	246, 252, 318	246.15
8	Deldrin	15.34	79, 108, 263	79.00
9	Triazophos	16.32	257, 172, 161	161.05
10	4,4, DDT	16.63	239, 235, 165	235.15
11	Deltametrin	16.73	181, 253, 251	181.05
12	Bifenthrin	17.32	182, 181, 166	181.10
13	Lambda-Cyhlaothrin	18.17	181, 197, 210	181.20

### 2.4 GC-MS method validation

The following parameters were validated: linearity, accuracy/precision, matrix effect, limit of detection (LOD), and limit of quantification (LOQ) according to SANTE/11945/2015 criteria [37]. After sample preparation and detection of 13 pesticides by GC-MS, the pesticide-free sample was chosen as the blank matrix sample. The field blank was not used in this study. Matrix-matched calibration curves were obtained by spiking at different concentrations (10, 25, 50, 100, 250, and 500 μg/kg) of mixed stock solution. Recovery and precision were determined based on the blank samples spiked at concentrations of 10 and 100 μg/kg in three replicates and held at room temperature for 30 min prior to use to ensure analyte-matrix interaction, and then handled according to the protocol stated in the sample preparation. The lowest detectable and quantifiable concentrations with a signal to noise ratio greater than 3 and 10 were estimated as LOD and LOQ, respectively.

To explore the influence of the co-extracted component on analytical signal intensity during GC-MS detection, of the matrix effect (ME) was investigated by comparing the signal intensity of matrix standard with that of a pure solvent standard at the same concentration. The matrix standard was created by dissolving the combined standard in a blank matrix solution. After dissolving the combined standard in acetonitrile, the matrix standard and solvent standard were injected into the GC-MS at the same concentration. Eq. (1) was used to calculate the ME.

(1) $$\mathrm{ME}\left(\%\right)=\frac{\mathrm{Slope}\;\mathrm{of}\;\mathrm{the}\;\mathrm{matrix}}{\mathrm{Slope}\;\mathrm{of}\;\mathrm{the}\;\mathrm{solvent}}-1*100$$

## 3 Results and discussion

### 3.1 Method development and validation

The SIM mode was used to conduct the analysis, which consisted of one target and two or three qualifier ions. Pesticides were characterized based on their retention time, monitored and confirmation ions. Table 2 summarizes the pesticides analyzed, along with their monitored and confirmation ions.

### 3.2 GC condition optimization

**Fig. 2:**
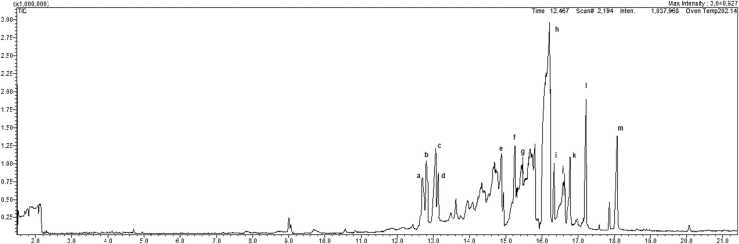
A representative chromatogram obtained for 13 pesticide standard a) pirimiphos-methyl, b)Fenitrothion, c) Malathion, d) Chlorpyrifos, e), Endosulfan, f) ethion, g) 4, 4, DDE, h) Deldrin, i) Triazophos, j) 4,4, DDT, k) Deltametrin, l) Bifenthrin, and m) Lambda-cyhlothrin at a SIM mode.

A GCMS-QP2010Plus chromatography of 13 pesticide standards analyzed is shown in Fig. 2. Each pesticide polarity is different, chromatographic columns and gradient elution were required to obtain a good chromatographic separation.

### 3.3 Linearity, LOD, and LOQ

The peak area was plotted against the concentration of the 13 pesticides at various calibration levels. All matrix-matched calibration curves were plotted using linear regression analysis. The coefficients of correlation (R^2^) were also computed, and all coefficients were greater than 0.99 for all pesticide residues, indicating good linearity (Table 3). Table 3 also includes the obtained LODs and LOQs, which show that the analytical approach has high sensitivity.

**Table 3:** Calibration data (regression coefficient, limit of detection and quantification) of 13 pesticide in spiked tea calibration curves.

No	Compound	Regression coefficient (R^2^)	LOD (μg/kg)	LOQ (μg/kg)
1	Pirimiphos- methyl	0.9985	0.48	1.45
2	Fenitrothion	0.9968	0.43	1.30
3	Malathion	0.9930	0.33	1.00
4	Chloropyrifos	0.9978	0.57	1.74
5	Endosulfan	0.9967	0.27	0.84
6	Ethion	0.9973	0.46	1.39
7	4,4, DDE	0.9991	0.12	0.36
8	Deldrin	0.9998	0.68	2.06
9	Triazophos	0.9994	0.84	2.55
10	4,4, DDT	0.9951	0.29	0.87
11	Deltametrin	0.9980	0.33	1.01
12	Bifenthrin	0.9937	0.45	1.36
13	Lambda-Cyhlaothrin	0.9941	0.33	0.99

### 3.4 Matrix effect (MEs)

**Fig. 3:**
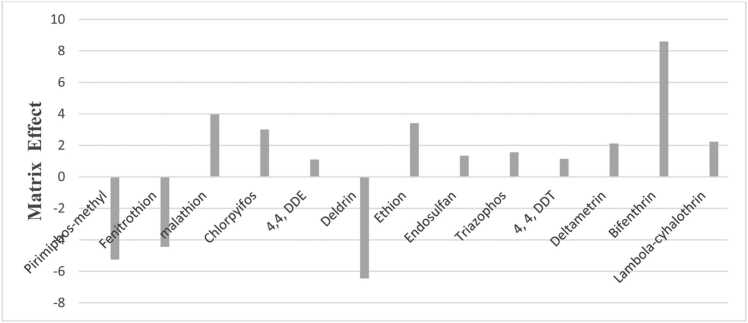
Matrix effect of the pesticide analyzed.

Matrix effects (MEs) are described as the aggregate influence of all sample components other than the analyte on the quantity measurement [41]. It is most common in chromatographic analysis, particularly with mass spectrometry. Even though many authors have investigated ME, the phenomenon of this mechanism is difficult to explain [26], [35], [29]. Various matrices will give different ME, even if they fit into the same group as stated by the SANTE/11945/2015 guidance document [12]. Based on ME % ranges, the percentage of ME can be positive or negative, and it's divided into three kinds of ME (low, medium, and strong matric effect) [19]. Low matrix effect is indicated as ME % values between − 20 and + 20; medium matrix effect is indicated as ME % values between –50 and − 20; + 50, and + 20. If ME% is higher − 50 and + 50, then it is considered a strong matrix effect. If the percentage difference between these slopes was positive, there would be a signal enhancement. It would indicate signal suppression if it was negative [19]. In this study, all pesticides had a low matrix impact, ranging from − 6.45–8.6 (Fig. 3), indicating that the optimized extraction technique and analytical conditions were in good accordance and satisfactory.

### 3.5 Recovery and reproducibility

Table 4 demonstrates the recovery and repeatability for two concentrations. Pesticide recovery ranged from 72% to 106%. Both analytes had suitable relative standard deviation (RSD) values of less than 20% in terms of repeatability. Taken together, these observations suggest that this approach fulfilled the SANTE/11945/2015 guideline's accuracy and precision requirements.

**Table 4:** Recovery and relative standard deviation (RSD) of the pesticides obtained from GC-MS analysis of tea samples at 3 spiking levels (n = 3).

Compound	Recovery% Spiked 10 (μg/kg)	RSD	Recovery %Spiked 100 (μg/kg)	RSD
Pirimiphos- methyl	94	2.10	90	4.42
Fenitrothion	82	8.05	98	1.16
Malathion	84	4.03	97	1.18
Chloropyrifos	89	3.32	88	2.46
Endosulfan	106	2.18	72	1.04
Ethion	84	1.95	95	2.81
4,4, DDE	97	3.28	100	1.00
Deldrin	91	1.78	93	2.29
Triazophos	92	4.61	87	2.03
4,4, DDT	94	2.99	95	2.29
Deltametrin	104	2.44	99	3.58
Bifenthrin	98	3.73	87	6.23
Lambda-Cyhlaothrin	74	2.02	96	1.55

### 3.6 Pesticide residues in the tea samples

The GC-MS method was successfully used to investigate pesticide residues in 100 rooibos tea samples in South Africa. The matrix effect was overcome in this study using spiked calibration curves, and the pesticides were monitored in tea samples after the analytical technique was validated. Pesticides were detected based on their retention time, and then confirmed by comparing them with that of standards. The reproducibility of recovery results indicated that the extraction and cleanup procedures were accurate enough for tea research (Table 4). Analysis of 100 samples showed the presence of deltamethrin and lambda- cyhlaothrin in only one of the samples, with a concentration of 92.11 and 66.41 μg/kg, respectively, which was found below the MRLs. None of the samples had residues of pirimiphos-methyl, fenitrothion, malathion, endosulfan, ethion, 4, 4, DDE, triazophos, 4, 4, DDT, deldrin, triazophos, chlropyiofos, and bifenthrin. Although the presence of deltamethrin and lambda- cyhlaothrin in only one of the samples does not imply that they are toxic, but long-term ingestions of low levels of pesticide residues that may accumulate in body organs/tissues over time may pose a possible health risk.

Results showed that analyzed pesticides were not present in all the tea samples. However, the presence of deltamethrin and lambda –cyhlaothrin may be a cause for concern and periodic monitoring should be needed. In an investigation conducted by Pitoi et al. [35], the concentrations of lambda-cyhalothrin, cypermethrin, fenvalerate, and deltamethrin in dried tea leaves were detected in their studies and were all below the MRLs, which is in agreement with the current study. In another similar study in China, specifically on tea marketed in the Tehran market, their results revealed that consumed tea in Iran is free of pesticide residues or has residues that are lower than existing MRLs [4], [45]. Natural factors such as temperature, rainfall, volatilization, photolysis, ventilation, biodegradation, pH, dew, and growth dilution, as well as the pre-harvest period between the last application and harvesting, can cause pesticide residues in tea bushes to degrade [39], [32], [44], [40]. In South Africa, the demand for organic farming has increased as customers become more health conscious and the role of food in maintaining a healthy lifestyle, which has contributed to the low use of chemicals in agriculture [43]. According to the European Union [16], over 486 pesticides have been regulated for tea in order to ensure safety and proper monitoring of the pesticide residues in tea. The present study has demonstrated a high level of conformity with global food safety standards and established criteria. Despite this high level of conformity, there is a need for further investigations into pesticide residues in rooibos teas in South Africa to ensure the consumer’s safety.

## 4 Conclusion

The presence of 13 pesticides in rooibos tea was assessed using an accurate, precise, and repeatable method. The process, which includes QuEChERS sample preparation and GC-MS-SIM analysis, demonstrated the high sensitivity and confirmatory capacity needed for determining pesticide residue at low levels. The excellent process validation findings demonstrated that the used technique for determining and quantifying pesticide residue in tea and other matrices is accurate, useful, and suitable. In the present study, only deltamethrin and lambda cyhalothrin were detected at concentrations below MRL. The findings of this study, on the other hand, highlight that rooibos tea in South Africa is safe with respect to pesticides analyzed, but there is still a need to investigate more of the pesticides used on tea fields and regular monitoring of pesticide residues is important for public health safety.

In future research, we will continue to investigate other regulated pesticides on rooibos tea and also present the research outcome to the regulatory policy in order to establish maximum residue limits on rooibos tea.

## CRediT authorship contribution statement

**OM. Areo:** Visualization, Writing – original draft, Writing – review & editing. **PB. Njobeh:** Funding acquisition, Project administration, Supervision, Writing – review & editing. **JO. Olowoyo:** Project administration, Supervision, Writing – review & editing. **LS. Sethoga:** Visualization, Conceptualization, Writing – original draft, Writing – review & editing. **OA Adebo:** Visualization, Conceptualization, Writing – original draft, Writing – review & editing.

## Declaration of Competing Interest

The authors declare that they have no known competing financial interests or personal relationships that could have appeared to influence the work reported in this paper.
